# Bedrohungsmanagement: deeskalieren, bevor etwas passiert

**DOI:** 10.1007/s11757-021-00673-w

**Published:** 2021-07-21

**Authors:** Angela Guldimann, Reinhard Brunner, Elmar Habermeyer

**Affiliations:** 1 Fachstelle Forensic Assessment & Risk Management (FFA), Klinik für Forensische Psychiatrie, Psychiatrische Universitätsklinik Zürich, Lenggstraße 31, Postfach 363, 8032 Zürich, Schweiz; 2 Präventionsabteilung, Kantonspolizei Zürich, Zürich, Schweiz

**Keywords:** Risikoeinschätzung, Gefährder, Querulant, Forensische Einschätzung, Behörden, Fürsorgepflicht, Risk assessment, Person of concern, Querulous person, Forensic assessment, Public authorities, Duty of care

## Abstract

In diesem Beitrag wird die Arbeit des Kantonalen Bedrohungsmanagements (KBM) Zürich vorgestellt. Personen, die durch ihre Kommunikation und/oder ihr Verhalten Hinweise auf ein mögliches Gewaltpotenzial zeigen (sog. Gefährder), sollen frühzeitig erkannt, eingeschätzt und so risikobehaftete Entwicklungen entschärft werden. Forensische Fachpersonen der Fachstelle Forensic Assessment & Risk Management (FFA) der Psychiatrischen Universitätsklinik Zürich unterstützen die polizeilichen Gewaltschützer darin, ein tragfähiges Fallverständnis im Hinblick auf die (psychisch kranken) Gefährder zu erarbeiten. Das Fallverständnis gilt es, im Rahmen von Gefährderansprachen sorgfältig zu überprüfen. In dieser Arbeit wird zudem erläutert, wie das KBM Behörden und Institutionen in der Einschätzung und im Management mit möglichen Gefährdern unterstützt. Hierbei werden potenzielle Fallstricke der Behördenmitglieder im Umgang mit Querulanten reflektiert, aber auch die Fürsorgepflicht des Arbeitgebers bzw. der betroffenen Behörden in den Fokus gerückt. Zuletzt werden auch Gefahren des Bedrohungsmanagementansatzes reflektiert, und es wird für höchstmögliche Transparenz gegenüber den potenziellen Gefährdern sowie den Bürgern und Bürgerinnen plädiert.

## Abstract (en)

This article presents the work of the Cantonal Threat Management (KBM) Zurich. People who, through their communication and/or behavior, give rise to serious concerns about their potential for violence (so-called person of concern) should be identified and assessed at an early stage. As a result, risky developments can be defused. Forensic specialists from the forensic assessment and risk management (FFA) department at the Psychiatric University Clinic in Zurich support the violence protection police officers in developing a sound case understanding, often in connection with persons who are mentally ill. The understanding of the case must be carefully checked when addressing the person of concern. This paper also explains how the KBM supports authorities and institutions in assessing and managing potential persons of concern. Potential pitfalls of members of authorities in dealing with querulous persons are reflected on but the employer’s duty of care is also brought into focus. Finally, the dangers of the threat management approach are also reflected and the plea is for the greatest possible transparency towards potential persons of concern and citizens in general.

## Bedrohungen erkennen, einschätzen und entschärfen

**Figure 1:**
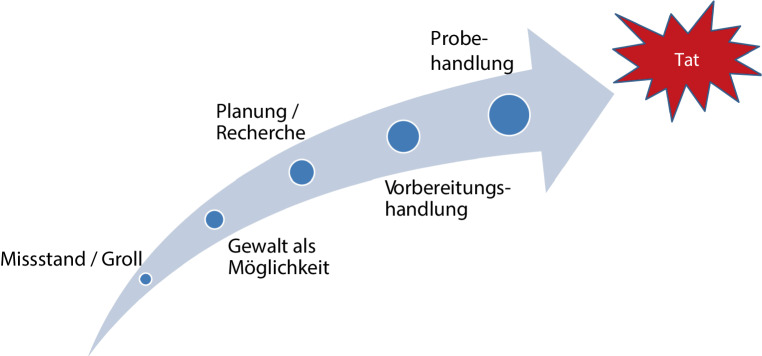

Die Geschichte des Bedrohungsmanagements, auf Englisch „threat assessment“ oder „threat management“, beginnt in den 1980er- und 1990er-Jahren in den USA. Damals wurden Attentate auf Personen des öffentlichen Lebens sowie der Versuch, solche Angriffe durch Sicherheitsbehörden zu verhindern, unter dem Begriff der *schweren zielgerichteten Gewalt *untersucht (Borum et al. 1999). Zielgerichtete Gewalt ist der Endpunkt eines grundsätzlich nachvollziehbaren Weges von Gedanken und Handlungen einer Person. Sie stellt aus deren subjektiven Sicht gleichsam die Lösung eines Missstands/Grolls oder einer Krise dar (Abb. 1; Herleitung: Calhoun und Weston 2003).

Von außen kann ein solcher Weg hin zu einer Gewalttat oftmals aufgrund von Warnsignalen erkannt werden (Meloy et al. 2012; Guldimann et al. 2013). Damit eröffnet sich grundsätzlich die Möglichkeit, im Rahmen eines Bedrohungsmanagements frühzeitig und präventiv einzugreifen (Allwinn und Hoffmann 2016). Ein Bedrohungsmanagement ist dann erfolgreich, wenn durch das rechtzeitige Erkennen, Einschätzen und Entschärfen von risikobehafteten Entwicklungen die tatsächliche Ausübung von Gewalttaten verhindert werden kann (Meloy et al. 2021). Dazu muss die Aktivierungsschwelle des Bedrohungsmanagements niedrig angesetzt werden, damit mögliche Bedrohungen frühzeitig erkannt werden, noch bevor die Schwelle zu einer Straftat überschritten ist. Ein Bedrohungsmanagement ist bereits dann angezeigt, wenn begründeter Anlass zu ernsthaften Befürchtungen in Bezug auf ein Gewaltpotenzial (Brunner 2017) besteht.

Der niederschwellige und präventive Ansatz des Bedrohungsmanagements unterscheidet dieses teilweise von der traditionellen kriminalprognostischen Vorgehensweise (Meloy et al. 2021). Diese befasst sich mit Risikoeinschätzungen im Rahmen von z. B. Strafverfahren und klärt in einem befristeten Zeitraum mittels Gutachten in der Regel auf die langfristige Kriminalprognose (d. h. auf einen Zeitraum von einem bis 3 Jahren) abzielende Fragestellungen wie beispielsweise die Möglichkeit der Haftentlassung einer beschuldigten oder verurteilten Person ab (Hoffmann und Roshdi 2015; Mokros et al. 2021). Demgegenüber befasst sich das Bedrohungsmanagement mit bedrohlichen Personen, die in der Regel in Freiheit sind und über die oft (noch) wenige Informationen verfügbar sind. Die Fallkonstellationen sind hier ausgesprochen dynamisch, d. h., es geht oftmals um Aussagen zum kurzfristigen, d. h. sich innerhalb von Tagen und Wochen manifestierenden Risiko einer Gewalthandlung. Die Fallbearbeitung wird inhaltlich und zeitlich weitgehend von dieser Dynamik bestimmt, d. h., die Situation muss bis zur Entschärfung der Lage in enger, interdisziplinärer Zusammenarbeit verschiedener Berufsgruppen (Polizei, Sozialarbeiter usw.) fortwährend neu beurteilt werden. Risikoeinschätzungen müssen dynamisch und, wenn nötig, innert Stunden erstellt werden, weil für ein präventives Eingreifen u. U. rasche Maßnahmen notwendig sein können.

Die Disziplin des Bedrohungsmanagements hat sich inzwischen weltweit etabliert (Meloy et al. 2021). Sie umfasst nicht mehr nur den Schutz von Personen des öffentlichen Lebens, sondern auch von Privatpersonen und Mitgliedern von Behörden sowie von Unternehmen, Schulen und Universitäten. Auch inhaltlich wird der Wirkungsbereich des Bedrohungsmanagements heute breit gefasst. Es geht, wie Hoffmann und Roshdi (2015) schreiben, *„nicht mehr alleine um die Verhinderung von schwersten Gewalttaten, sondern auch um den Umgang mit einem breiteren Spektrum bedrohlicher Verhaltensweisen, die zu schweren Belastungen bei den Betroffenen führen können“ *(S. 267).

In diesem Beitrag wird die Arbeit des Bedrohungsmanagements am Beispiel des Kantons Zürich vorgestellt.

## Kantonales Bedrohungsmanagement

Der Einführung des Kantonalen Bedrohungsmanagements (KBM) in Zürich ging ein zweifaches Tötungsdelikt voraus, das ein breites mediales Echo fand: Im August 2011 wurden eine in Trennung lebende Frau und Mutter von 6 Kindern sowie die Leiterin des für die Familie zuständigen Sozialamtes durch den gewalttätigen und behördlich bereits bekannten Ehemann mit Kopfschüssen auf offener Straße getötet. Diese Tat war der Anlass für zahlreiche politische Vorstöße, um die Gewaltprävention auf mehreren Ebenen zu verbessern, u. a. durch eine bessere rechtliche Abstützung der Zusammenarbeit zwischen Behörden (Brunner 2017).

Bei der Polizei wurde ein Paradigmenwechsel eingeleitet: weg von einer rein repressiven Polizeiarbeit hin zu einer präventiven, d. h. vorausschauenden Polizeiarbeit (Brunner 2017). Es wurden 3 spezialisierte *Gewaltschutzstellen *bei der Kantonspolizei sowie bei den beiden Stadtpolizeien Zürich und Winterthur geschaffen. Bei diesen Fachstellen kann nun seit 2012 jeder Bürger und jede Bürgerin des Kantons Zürich als Privatperson oder in der beruflichen Funktion niederschwellig Unterstützung in bedrohlichen Situationen und bei der Einschätzung von Gewaltrisiken erhalten. Kritische, noch wenig auffällige Konstellationen (z. B. familiäre Differenzen ohne Anzeige) werden oftmals als Erstes auch von den polizeilichen Frontkräften in den Regionen erkannt. Ihnen steht der Gewaltschutz intern für Fallbesprechungen zur Verfügung. In den Gewaltschutzstellen arbeiten spezialisierte polizeiliche Mitarbeitende, die sich der vielfältigen Gefährdungs- und Bedrohungssituationen gezielt und, wann immer möglich präventiv, annehmen. Sie befassen sich dabei mit einem breiten Spektrum von Gewaltsituationen, von Familienstreitigkeiten, häuslicher Gewalt, Stalking, (Amok‑)Drohungen und Querulanz bis hin zu Radikalisierung und terroristischen Aktivitäten.

Jede Meldung wird kritisch dahingehend überprüft, ob die gemeldete Person überhaupt als *Gefährder* eingestuft werden kann. Eine national oder international verbindliche wissenschaftliche oder rechtliche Definition des Begriffs Gefährder existiert gegenwärtig nicht (Greuter 2017). Die Präventionsabteilung des Gewaltschutzes der Kantonspolizei Zürich (2020)^1^ definiert Gefährder wie folgt: „Als Gefährder/in gelten Personen, die durch ihr Verhalten und/oder ihre Äusserungen (Warnsignale) begründet Anlass zu ernsthaften Befürchtungen geben, dass sie in absehbarer Zeit eine Gewalttat gegen die physische, psychische und/oder sexuelle Integrität zum Nachteil von Dritten begehen könnten und diese dadurch in ihrer Handlungsfreiheit beeinträchtigen (Gefährdungssituation).“

In Rahmen der Prüfung werden Äußerungen (z. B. „Ich verstehe, warum jemand Amok läuft“) und Verhaltensweisen (z. B. Privatadressen ausfindig machen) der gemeldeten Person im Gesamtkontext (z. B. Kündigung durch Arbeitgeber erhalten) evaluiert. Die Gewaltschützer erheben dazu in einem standardisierten Verfahren Informationen zum besorgten Melder, zu dessen Beweggründen für die Meldung und zu seiner Beziehung zum möglichen Gefährder. Zudem werden Informationen über die Gesamtsituation, den potenziellen Gefährder und evtl. gefährdete Personen erfragt. Aufgrund dieser Informationen nehmen die Gewaltschützer eine Erstbeurteilung vor und veranlassen, wenn nötig, erste konkrete Maßnahmen zur Gefahrenabwehr. Eine besondere Herausforderung stellt sich den polizeilichen Gewaltschutzstellen, wenn Gefährder schwere psychische Auffälligkeiten aufweisen, da sie selbst nicht über psychiatrisch/psychologisch-forensisches Fachwissen im Umgang mit solchen Personen verfügen.

Die in dieser Ausgabe präsentierten Erkenntnisse von Lorey und Fegert (2021) unterstreichen die vom Zürcher Gewaltschutz angesprochenen Problemfelder im Umgang mit psychisch auffälligen Personen: In einer fragebogengestützten Untersuchung von über 2000 Polizeibeamten aus Baden-Württemberg gab die Mehrheit der Befragten an, dass die größte Herausforderung im Arbeitsalltag der Polizei in der Beurteilung der Gefährlichkeit der psychisch auffälligen Personen sowie in der schwierigen Vorhersagbarkeit ihrer Verhaltensweisen bestehe. Die Befragten äußerten das klare Bedürfnis nach Fortbildung (50 %) sowie nach einer verbesserten Vernetzung mit professionellen Helfern (39 %). Die oben genannten Autoren plädieren für multidisziplinäre praxisorientierte Fortbildungsangebote und eine engere Kooperation u. a. zwischen Polizei und Forensik.

## Fachstelle Forensic Assessment & Risk Management

Der Ansatz der engen und interdisziplinären Kooperation wird im Rahmen des KBM seit 2014 in Zürich gelebt. Mit der Implementierung der *Fachstelle Forensic Assessment & Risk Management (FFA)* der Psychiatrischen Universitätsklinik Zürich wurde im KBM der niederschwellige Zugriff auf forensisches Fachwissen sichergestellt (Habermeyer und Guldimann 2019; Beyli-Helmy et al. 2020). Die FFA unterstützt die polizeilichen Gewaltschutzfachstellen, die Staatsanwaltschaften und die akutpsychiatrischen Versorgungsklinken im Kanton mit kurzfristigen Risikoeinschätzungen und forensischem Fachwissen im Risikomanagement (Schmidt et al. 2021). Der vorliegende Beitrag fokussiert auf die Zusammenarbeit mit dem polizeilichen Gewaltschutz.

In der FFA arbeiten (Rechts‑)Psychologen und forensische Psychiater. Einige Mitarbeiter ergänzen die FFA-Kompetenzen mit ihrer kriminologischen und juristischen Ausbildung. Die Einschätzung von unter 18-jährigen Gefährdern ist dank des Einbezugs des hiesigen Zentrums für Kinder- und Jugendforensik möglich. Um Lern- und Synergieeffekte zu fördern, hat die FFA Arbeitsplätze bei allen 3 Gewaltschutzstellen im Kanton Zürich bezogen. Im Arbeitsalltag finden regelmäßig Fallbesprechungen mit den Gewaltschützern, Fallkonferenzen mit beteiligten Behörden und insbesondere auch die Planung und Durchführung von *Gefährderansprachen* sowie von Gesprächen mit gefährdeten Personen und Angehörigen von Gefährdern statt. Im Weiteren nimmt die FFA eine Brückenfunktion zwischen den therapeutischen Behandlern der Gefährder und den polizeilichen Gewaltschützern ein. Hier ist es die Aufgabe der FFA, die verschiedenen Berufsgruppen mit ihren berufsbezogenen Fachsprachen, Sichtweisen, Erwartungen und Zuständigkeitsbereichen zu verbinden. Dabei „übersetzt“ die FFA beispielsweise psychiatrische Diagnosen und berät die Gewaltschützer, wie sie damit z. B. im Rahmen einer Gefährderansprache auf Polizeiebene umgehen können.

## Gefährderansprachen

Unter einer Gefährderansprache wird die in der Regel zeitnahe Kontaktaufnahme durch den Gewaltschutz mit den als Gefährdern eingeschätzten Personen nach einer Meldung verstanden. Mit Gefährderansprachen werden mehrere Ziele verfolgt (Greuel et al. 2010; Beyli-Helmy et al. 2020): 1) Informationsgewinn zur zuverlässigeren Beurteilung des Gewaltrisikos und für die Umsetzung von risikoreduzierenden Interventionen; 2) Aufzeigen, Vermittlung und/oder Umsetzung von risikoreduzierenden Unterstützungsmaßnahmen; dazu gehört auch die Klärung der Voraussetzungen für die weitere Zusammenarbeit mit dem Gewaltschutz; 3) Grenzziehung und Verdeutlichung der rechtlichen und gesellschaftlichen Verhaltensnormen.

Die Gefährderansprache hat also einerseits das Ziel, Informationen für eine breiter abgestützte Risikoeinschätzung zu generieren. Sie kann bestenfalls aber auch bereits eine risikoreduzierende Intervention darstellen. Die Risikoeinschätzung und das Risikomanagement lassen sich somit nicht klar voneinander trennen. Im Kanton Zürich basieren die Gefährderansprachen auf Freiwilligkeit. Sofern allerdings ein Gefährder bereits in ein Strafverfahren involviert ist, kann die Staatsanwaltschaft eine Auflage zur Zusammenarbeit mit dem Gewaltschutz als Ersatzmaßnahme verfügen.

Zeigen sich die Gefährder im freiwilligen Setting gesprächsbereit, besuchen die Gewaltschützer die Gefährder nach vorgängiger meist telefonischer Absprache zu Hause, am Arbeitsplatz oder an einem öffentlichen Ort. Dies erfolgt immer unter der Prämisse, dass aus polizeilicher Sicht keine Sicherheitsbedenken hinsichtlich der gewählten Örtlichkeit bestehen. Gefährderansprachen können als einmalige Intervention oder mehrfach erfolgen (Greuter 2017). Die Gewaltschützer führen die Gefährderansprachen in der Regel in zivil und zu zweit durch. Das Vier-Augen-Prinzip ermöglicht einerseits einen umfassenderen Blick auf den Gefährder. Andererseits muss auch der Gesundheit und Sicherheit aller Beteiligten Rechnung getragen werden. Die forensischen Fachpersonen der FFA begleiten die Gewaltschützer jeweils bei Hochrisikofällen, bei unklarem Gefährdungspotenzial, chronifizierten Fallverläufen und dem Verdacht auf eine psychische Erkrankung. Die beteiligten Berufsgruppen machen vor der Gefährderansprache ihre jeweilige Funktion gegenüber den Gefährdern transparent. Die Spezifika der jeweiligen Berufsgruppe können zum Beziehungsaufbau mit den Gefährdern genutzt werden: So kann der polizeiliche Gewaltschützer bei einem waffenaffinen Gefährder mit seiner Schusswaffenexpertise ein gegenseitiges Verständnis aufbauen, während die forensische Fachperson in Bezug auf Probleme in der psychotherapeutischen Behandlung oder mit Medikamentennebenwirkungen an die Erlebenswelt der Gefährder anknüpfen kann.

## Fallverständnis gemeinsam erarbeiten und kritisch überprüfen

Vor einer Gefährderansprache wird in einem ersten Schritt gemeinsam mit den Gewaltschützern ein Fallverständnis erarbeitet, welches während der Gefährderansprachen kritisch überprüft werden muss (Beyli-Helmy et al. 2020). Ein belastbares Fallverständnis bildet gemäß Cook et al. (2014) die Grundlage einer fundierten Risikoeinschätzung. In die Erarbeitung des Fallverständnisses fließen Wissensvermittlung (z. B. Erläuterung psychiatrischer Störungen), das Zusammentragen und die Bewertung der zu dem Zeitpunkt bekannten Risikofaktoren und der Verweis auf aus forensischer Sicht fehlende wichtige Informationen ein. Basierend auf dem Fallverständnis werden Gesprächstechniken vermittelt, die im Einzelfall einen erfolgreichen Erstkontakt ermöglichen sollen. Als konkrete Hilfestellung wird u. a. das Konzept der komplementären Beziehungsgestaltung nach Sachse (2020) herangezogen. Sachse (2020) nennt 6 Beziehungsmotive, die den Menschen in Beziehungen wichtig sind und mit ihrer Persönlichkeit im Zusammenhang stehen:

*Anerkennung* (das Bedürfnis, über die eigene Person positive Rückmeldungen zu erhalten), *Wichtigkeit *(das Bedürfnis, im Leben einer anderen Person eine wichtige und bereichernde Rolle zu spielen), *Verlässlichkeit *(das Bedürfnis, nach einer Beziehung, die berechenbar, beständig und belastbar ist), *Solidarität* (das Bedürfnis, Hilfe und Unterstützung zu bekommen, wenn es nötig ist), *Autonomie* (das Bedürfnis, eigene Entscheidungen zu treffen, eigene Lebensbereiche zu haben und als autonome Person bestehen zu dürfen) und *Grenzen* (das Bedürfnis, sein eigenes Territorium zu definieren, über seine Grenzen zu bestimmen und darüber zu entscheiden, wer hinter die Grenzen treten darf).

Durch die Beachtung der Beziehungsmotive kann ein sog. *Beziehungskredit* aufgebaut werden. Das Gegenüber fühlt sich wahrgenommen und verstanden, was zu einem tragfähigen Arbeitsbündnis mit den Gefährdern beiträgt, das in der Folge auch Kritik oder Konfrontation besser aushält. Bei Gefährdern mit einem hohen Bedürfnis nach Autonomie und Wahrung der Grenzen, wie z. B. bei Personen mit einer paranoiden Persönlichkeitsstörung, werden im Rahmen einer Gefährderansprache so viel Transparenz („Ich möchte Ihnen genau darlegen, worum es geht. Bitte haken Sie nach, wenn Fragen auftauchen“) und Entscheidungs- bzw. Kontrollmöglichkeiten zugestanden, wie es verantwortbar und unter den gegebenen Umständen realistisch ist („Ich zeige Ihnen Optionen auf, Ihnen aber obliegt es zu entscheiden“).

Neben dem Versuch, die Beziehungsmotive zu ergründen und daran anzuknüpfen, sind das Erkennen und Nutzen von Ressourcen und protektiven Faktoren bei Gefährdern im weiteren Risikomanagement wichtig. Die forensische Psychiatrie hat seit einigen Jahren ihren zuvor besonders auf Risikofaktoren fokussierten Blickwinkel um einen ressourcenorientierten Blickwinkel erweitert (Good Lives Model; Franqué und Briken 2013; SAPROF; de Vogel et al. 2009). Ressourcenorientierung bietet sich gerade im Setting des Bedrohungsmanagements an, da ein einseitiger Fokus auf Defizite nicht geeignet ist, eine tragfähige Arbeitsbeziehung zu entwickeln, umso mehr, als die Zusammenarbeit mit Gefährdern oft auf freiwilliger Basis etabliert werden muss. Andererseits eröffnet die Sicht auf die funktionierenden Lebensbereiche oder auf grundsätzlich positive Fähigkeiten, die im Rahmen des Problemverhaltens jedoch „destruktiv“ eingesetzt werden (z. B. Durchhaltevermögen als Stärke, aber im Rahmen von Querulanz destruktiv eingesetzt), Raum für risikomindernde Überlegungen. Es ist zudem anzunehmen, dass Gefährder (noch) ohne strafrechtliche Vorbelastung über mehr psychosoziale Ressourcen verfügen als solche, die eine jahrelange strafrechtliche Vorgeschichte aufweisen. Diese Ressourcen gilt es, zu erhalten und möglichst auch präventiv zu nutzen.

Nach erfolgter Gefährderansprache und ggf. unter Einbezug weiterer Informationen (z. B. Fremdanamnese durch die Behandler nach Entbindung von der Schweigepflicht) nimmt die FFA in der Regel schriftlich im Vier-Augen-Prinzip Stellung zum kurzfristigen Gewaltrisiko zuhanden des Gewaltschutzes. Dies erfolgt unter Nennung fehlender Informationen und Benennung von Unsicherheiten in der Einschätzung. Die FFA formuliert risikosenkende Maßnahmen und gibt Hinweise auf Anzeichen für risikoerhöhendes Verhalten im Risikomanagement. Idealerweise werden dabei standardisierte Instrumente für die Risikobeurteilung beigezogen. Dabei sind Instrumente, die auf dem Modell der strukturierten professionellen Risikobeurteilung basieren, z. B. HCR-20, Version 3 (Douglas et al. 2013) oder das Stalking Assessment and Management (SAM; Kropp et al. 2008), für die Bedürfnisse des Bedrohungsmanagements besonders gut geeignet, denn sie fördern bzw. erzwingen eine Szenarienbildung, die mit Interventionsstrategien verbunden werden kann. Auf diese Weise kann auch festgelegt werden, wie bei einer möglichen Eskalation reagiert werden kann und soll. Demgegenüber sind aktuarische Instrumente im Rahmen des Bedrohungsmanagements nur begrenzt hilfreich, da sie die dynamischen Risiko- und Schutzfaktoren des Gefährders sowie die Entwicklung verschiedener Risikoszenarien nicht berücksichtigen (Habermeyer und Guldimann 2019).

Die Anwendung spezifischer Instrumente ersetzt jedoch keinesfalls die individuelle Fall- und Risikoanalyse unter Berücksichtigung von Verhaltensmustern des Gefährders. Hierbei werden 8 empirisch untersuchte Warnverhalten berücksichtigt, die auf risikoerhöhende Verhaltensmuster der Gefährder auf dem Weg zur Gewalt hindeuten können (Guldimann und Meloy 2020): *1. Anzeichen der Fixierung* (z. B. Fixierung auf eine Person/Thema), *2. neu auftretende Formen der Gewalt* (z. B. noch nie zuvor gezeigte Aggressionshandlungen), *3. Identifizierung* (z. B. Glorifizierung von Waffen, Gewaltverbrechern, Ideologien), *4. Energieschub* (z. B. Intensivierung des Verhaltens), *5. „leakage“* (z. B. Kommunikation einer Tatabsicht gegenüber Dritten), *6. direkte Drohung* (z. B. direkte Ankündigung der Tatabsicht), *7. letzter Ausweg* (z. B. zunehmende Verzweiflung), *8. der Weg zur Gewalt* (z. B. konkrete Vorbereitungshandlungen).

Falls in der Risikoeinschätzung ein Gewaltpotenzial erkannt wird und ein Bedrohungsmanagement (weiter) indiziert erscheint, ist aus lerntheoretischen Überlegungen Folgendes zu beachten: Gefährder erhalten aufgrund ihres negativen Verhaltens (u. a. Drohungen aussprechen, Unzuverlässigkeit) von Behörden und Fachpersonen, inkl. Gewaltschutz und Forensik, intensive Aufmerksamkeit. Dies ist zwar richtig, um zur Deeskalation und Krisenintervention beizutragen. Das Reaktionsmuster darf aber nicht dazu führen, dass Gefährder lernen, dass v. a. negatives Verhalten zu Aufmerksamkeit oder gar Zuwendung führt. Unter lerntheoretischen Aspekten ist es wichtig, positives und lösungsorientiertes Verhalten der Gefährder (u. a. Absprachen einhalten, um Rat fragen) zeitnah mit Aufmerksamkeit zu verstärken, um die Auftretenswahrscheinlichkeit dieses Verhaltens zu erhöhen (Michael et al. 2018).

## Vom Individuum zum Gesamtsystem: Unterstützung von Behörden und Institutionen im KBM

An die vorstehend genannten, lerntheoretisch-interaktionellen Gedanken anknüpfend sind auch systemische Überlegungen relevant: Ein Gefährder ist als Individuum auf vielfältige Weise in verschiedene Systeme eingebettet, in denen das bedrohliche Verhalten unterschiedlich stark zutage treten kann. Diese unterschiedlichen Systeme gilt es, miteinander abzustimmen bzw. die dort gemachten Erfahrungen in ein Fallkonzept zu überführen. Letztlich geht es, bildlich gesprochen, um kommunizierende Röhren, die nicht getrennt voneinander betrachtet werden können.

Im Kanton Zürich wurde zur Unterstützung der Behörden und Institutionen in den letzten Jahren ein Netz mit *Ansprechpersonen* implementiert (von Rohr et al. 2013; Brunner 2017). Diese Ansprechpersonen sind Mitarbeitende von Behörden oder Institutionen (u. a. Gemeinden, Spitäler, Opferhilfestellen, Kindes- und Erwachsenenschutzbehörden, Gerichte, Schulen, Universitäten etc.). Sie fungieren intern als Ansprechperson für als bedrohlich wahrgenommene Kunden/Klienten und (Ex‑)Mitarbeitende.

Die Ansprechpersonen stellen im Alltag das Bindeglied zu den polizeilichen Gewaltschutzstellen dar, um im konkreten Einzelfall einen behördenübergreifenden strukturierten und harmonisierten Informationsfluss zu gewährleisten. Die Ansprechpersonen sind in der Lage, eine erste Bewertung vorzunehmen. Sie können den Fall jederzeit und besonders beim Vorliegen einer möglichen Risikokonstellation anonymisiert mit dem zuständigen Gewaltschutzdienst besprechen. Besonders schützenwerte Personendaten werden basierend auf den entsprechenden Datenschutzgrundlagen erst dann übermittelt, wenn die Schilderungen eine vertiefte Abklärung der gemeldeten Person bzw. Situation notwendig erscheinen lassen, um notwendige risikosenkende Maßnahmen treffen zu können (Brunner 2017). Die Ansprechpersonen werden in der Regel jährlich von den Gewaltschützern, dem polizeilichen Rechtsdienst, den Mitarbeitenden der FFA sowie weiteren Experten geschult. Die Weiterbildung umfasst u. a. die Erkennung von Risikokonstellationen, datenschutzrechtliche Fragen und den konkreten Umgang mit psychisch auffälligen Personen. Aktuell nehmen im Kanton Zürich knapp 600 Personen eine Funktion als behördeninterne Ansprechperson wahr.

## Praxisbeispiel „Querulanz“

Der Begriff „Querulant“ hat lateinische Wurzeln (*queri* *=* *vor Gericht klagen*), ist kein medizinischer Fachbegriff, sondern wurde anfänglich von Juristen zur Identifikation von „Streitsuchenden“ und „Prozesskrämern“ geprägt (Lindemann 2014). Die Abgrenzung von (schwierigen) Beschwerdeführern zu (gewalttätigen) Querulanten erfolgt entlang einem Kontinuum. Einerseits sind die Merkmale „Ungerechtigkeitserleben“ und „Persistenz beim Beschwerdeführer“ nicht ausreichend, um bereits von querulatorischem Verhalten zu sprechen (NSW Ombudsman 2021), andererseits steht Querulanz nicht automatisch mit einem Gewaltrisiko im Zusammenhang (Rossegger et al. 2012). Aus diesem Grund muss im konkreten Fall stets reflektiert werden, ob es sich um einen kritischen Bürger oder eine kritische Bürgerin handelt, der/die sein/ihr Recht auf freie Meinungsäußerung wahrnimmt und wahrnehmen darf, oder ob es sich um querulatorisches Verhalten, evtl. vor dem Hintergrund einer schweren psychiatrischen Erkrankung mit erhöhtem Gewaltpotenzial, handelt (Sass 2010).

Ein Blick auf die aktuellen Zahlen der polizeilichen Kriminalstatistik PKS Schweiz 2020 zeigt die Relevanz des Themas auf: So erreichte der Tatbestand „Gewalt und Drohung gegen Behörden und Beamte“ 2020 einen Höchststand. Mit 3514 Straftaten resp. einer Zunahme von 8,1 % gegenüber dem Vorjahr, wurden so viele Verzeigungen wie noch nie in den letzten 10 Jahren verzeichnet (Bundesamt für Statistik 2020). Es ist anzunehmen, dass die durch die COVID-19-Pandemie bedingten rechtsstaatlichen Eingriffe in das Leben der Bürger und Bürgerinnen einen Beitrag beim Anstieg von Gewalt und Drohungen gegen Behörden und Beamte geleistet haben.

Für Behördenmitglieder stellt der Umgang mit Drohern und Querulanten eine besondere Herausforderung dar; dies deshalb, weil eine Behörde nicht nur mit Fällen von Querulanz umgehen muss, sondern selbst Partei in der konkreten Fallkonstellation ist oder sein kann. Dazu schreibt Lindemann (2014), dass „der berechtigte Hinweis der Soziologie auf den nicht unerheblichen Eigenanteil des Justizsystems am Zustandekommen von Querulanz nichts daran ändert, dass es sich bei der intensiven Beanspruchung der Gerichte durch einige wenige Rechtssuchende um ein reales Phänomen handelt (S. 145)“. Letztlich bleibt es, unabhängig von der Vorgeschichte dabei, dass die betroffenen Behörden sowohl die Beschwerdeflut professionell handhaben als auch weiterhin einen geregelten Ablauf des Betriebs sicherstellen müssen. Die involvierten Mitarbeiter treffen in den Beschwerdeführern teils auf psychisch auffällige, z. T. sogar schwer erkrankte Personen mit intensivem (tatsächlichem oder infolge ihrer Psychopathologie auftretendem) Ungerechtigkeitserleben, hoher Beratungsresistenz und wenig Spielraum für Lösungsansätze. Die Behördenmitglieder befürchten oft eine gewalttätige Eskalation sowie einen medialen Reputationsschaden, insbesondere wenn im Vorfeld tatsächlich Fehler in der Fallbehandlung erfolgt sind. Diese Faktoren tragen dazu bei, dass querulatorisches Verhalten die Behördenmitglieder auch emotional stark belasten kann (Lester 2017).

Im Einzelfall ist es deshalb wichtig, einen genauen Blick auf die Fallkonstellation zu werfen und auch die behördeninternen Prozesse, die Kommunikation sowie die Reaktion der Behördenmitglieder zu analysieren, um ein umfassendes Fallverständnis zu bekommen. Eine Eskalationsspirale aus Aktion und Reaktion zwischen Behörde und Querulant sollte rasch unterbrochen bzw. soweit möglich reduziert werden.

## Selbstreflexionsfragen

Um einer ungünstigen Dynamik vorzubeugen, muss das eigene Handeln kritisch reflektiert werden. Die folgenden Selbstreflexionsfragen dienen in der Praxis als Hilfestellung beim Erkennen von Konstellationen, die nicht zuletzt die emotionale Belastung der Mitarbeitenden vergrößern können. Sie sind teilweise auch von Lester (2017) und der NSW Ombudsman Stellen (2021) als mögliche Fehlerquellen im Umgang mit unangemessenem Verhalten von Beschwerdeführern und Querulanten beschrieben und aus den praktischen Erfahrungen der FFA ergänzt worden. Die Reflexionsfragen werden im Rahmen von Schulungen vermittelt. Dem Coaching von Führungspersonen kommt gemäß Schulze (2010) im Bedrohungsmanagement auch deswegen eine hohe Bedeutung zu, damit diese in der Lage sind, ihre Fürsorgepflicht gegenüber den Mitarbeitenden wahrzunehmen.

Die Selbstreflexion soll auf 3 Ebenen erfolgen:

*Behördeninterne Strukturen, Prozesse und Werthaltungen:*

- Wird der Umgang mit „Beschwerden/Querulanz“ als Führungsaufgabe verstanden oder an Mitarbeiter delegiert?
- Ist das Konzept der prozeduralen Gerechtigkeit bekannt? Werden Beschwerdeführer grundsätzlich ernst genommen und ihre Beschwerden anhand eines nachvollziehbaren und behördenintern etablierten Prozesses bearbeitet?
- In welchem Zeitrahmen kann mit einer Antwort auf eine Beschwerde gerechnet werden? Erhält ein Beschwerdeführer eine Eingangsbestätigung?
- Wird dem Beschwerdeführer auch aktiv Rückmeldung über den Stand der Bearbeitung gegeben?
- Ist die Behörde bereit, komplizierte Prozesse oder schwierige Sachverhalte zu vereinfachen, sodass weniger Missverständnisse entstehen?
- Nutzt die Behörde das Konzept der „leichten Sprache“, um schwierige Sachverhalte allgemeinverständlich zu formulieren?
- Wie steht es um die Fehlerkultur in der Behörde? Welche Reaktion haben Mitarbeiter, aber auch Vorgesetzte zu erwarten, wenn ein Versäumnis unterlaufen ist oder ein Fehler begangen wurde?

*Unterstützung im konkreten Einzelfall:*

- Sind genügende Ressourcen für die Fallbearbeitung vorhanden bzw. werden diese bereitgestellt?
- Werden die mit dem Fall befassten Behördenmitglieder von ihren Vorgesetzten und Arbeitskollegen unterstützt, oder wird die „heiße Kartoffel“ intern weitergereicht?
- Werden Antwortschreiben von Kollegen kritisch gegengelesen, oder werden die betroffenen Mitarbeiter allein gelassen („Mach einfach“)?
- Wurden seitens der Behörden Fehler begangen? Liegen berechtige Gründe vor, vom normalen Prozedere in der Behörde abzuweichen? Ist die Behörde willens, sich auf pragmatische Lösungen einzulassen, oder besteht die Befürchtung von Reputations- und Haftungsschäden? Ist die Behörde bereit, unkonventionelle und pragmatische Wege zu gehen?

*Selbstreflexionsfragen für Behördenmitglieder:*

- Habe ich als Behördenmitglied verstanden, worum es dem Beschwerdeführer/Querulanten wirklich geht? Achtung: Die Anliegen von psychisch schwer kranken Personen sind teils nicht (mehr) nachvollziehbar.
- Welche Gefühle lösen der Beschwerderführer/Querulant oder seine Schreiben bei mir aus? (Verärgert, mitfühlend, ängstlich …)?
- Bin ich persönlich im Fokus des Beschwerdeführers/Querulanten? Lasse ich mich bei meinen Arbeitsschritten von (zu vielen) Emotionen leiten?
- Stimme ich in meiner persönlichen Haltung mit der Beschwerde/dem Anliegen des Beschwerdeführers/Querulanten überein (z. B. Kritik an staatlichen Anticoronamaßnahmen)? Beeinflusst meine persönliche Haltung meine Arbeitsschritte als Behördenmitglied?
- Was würde ich einem Beschwerdeführer ohne querulatorische Aktivitäten auf seine Beschwerde antworten? Welche Gründe gibt es, hier evtl. anders zu antworten?
- Schiebe ich Antwortschreiben aus Frust oder Angst auf, was zu langen Antwortreaktionen beiträgt? Oder bin ich überbemüht, zeitnah evtl. (zu) rechtfertigende oder (zu) detaillierte Antworten zu verfassen, um die Anspruchshaltung des Beschwerdeführers/Querulanten zu erfüllen?
- Zu welchem Zeitpunkt delegiere ich als Behördenmitglied den Beschwerdeführer/Querulanten an meine vorgesetzte Stelle für eine Stellungnahme? Folge ich dabei einem definierten Prozess oder reagiere ich auf Druck des Beschwerdeführers/Querulanten, weil dieser eine Überprüfung wünscht? Welche Botschaft sende ich mit dieser Handlung? Wird er so in seinem Anliegen ernst genommen? Oder wird v. a. sein druckausübendes Verhalten mit Aufmerksamkeit durch die nächsthöhere Stelle „belohnt“?

Die kritische Selbstreflexion hilft den Behörden, ihr Handeln gegenüber außen wie auch innerhalb der Organisation zu objektivieren und ggf. zu verbessern. Es wirkt auf einige Behördenmitglieder entlastend, wenn ihnen aus psychologischer Sicht erläutert wird, dass ihre Reaktion auf die schwierige Situation (z. B. sich nicht mehr mit den Eingaben/Beschwerden befassen zu wollen) normalpsychologisch nachvollziehbar ist oder auch der Hinweis aus Sicht der FFA/Gewaltschutz erfolgt, dass der Fall bisher gut gemanagt wurde.

## Was ist ein erfolgreiches Bedrohungsmanagement?

Wie andere Behörden muss auch das Bedrohungsmanagement seine eigene Arbeitsweise reflektieren. Der vorab beschriebene präventive Ansatz steht grundsätzlich und besonders im Rahmen von besonderen gesellschaftlichen Entwicklungen wie einer Pandemie mit rechtstaatlichen Eingriffen in die Autonomie der Bürger in der Pflicht, kritischen, impulsiven, hartnäckigen Menschen nicht leichtfertig das Label „Gefährder“ anzuheften (Simmler und Brunner 2021). Die Bedrohungsmanager müssen sich der Unsicherheiten und Grenzen von Verhaltensvorhersagen zudem bewusst sein und ausdrücklich darauf hinweisen.

Ein weiteres grundlegendes Prinzip ist das der Transparenz: Im Gegensatz zur kriminalpolizeilichen Ermittlung, die zur Beweissammlung und -sicherung größtenteils im „Verborgenen“ erfolgt, sieht das Bedrohungsmanagement eine offene Herangehensweise vor. Diese wird zunächst über eine entsprechende Öffentlichkeitsarbeit sichergestellt. Um adäquat über die Funktion und die Zielsetzungen des Bedrohungsmanagements zu informieren, hat z. B. die Kantonspolizei Zürich eine Webseite zum KBM aufgeschaltet (www.kbm.zh.ch). Die Transparenz gilt jedoch auch für Betroffene, denn die Person, von welcher mutmaßlich eine Gefahr ausgeht, soll bzw. muss wissen, dass ihr problematisches Verhalten erkannt worden ist, und dass dagegen etwas unternommen wird. Insofern wird das Vorgehen im Bedrohungsmanagement, was im Rahmen der Gefährderansprachen besonders deutlich wird, auch gegenüber möglichen Gefährdern offengelegt. Die praktischen Erfahrungen zeigen, dass dieses Vorgehen präventive Wirkung entfaltet. Nicht zuletzt Gefährder, die mit Behörden im Konflikt stehen, schätzen es, wenn ihnen Gehör gewährt wird. Nicht selten ist die Aussage zu hören: „Endlich hört mir mal jemand richtig zu“.

Die Messung der Quantität und Qualität der Gewaltdelikte, die durch ein erfolgreiches Bedrohungsmanagement verhindert werden, stellt jedoch wie auch in anderen Bereichen der Gewaltprävention eine besondere Herausforderung dar. In den letzten Jahren wurde zunehmend versucht, die Präventionswirkung des Bedrohungsmanagements empirisch fassbar zu machen. James und Farnham (2016) konnten zeigen, dass bei 100 mehrheitlich psychisch kranken Gefährdern durch u. a. freiwillige und unfreiwillige Klinikeintritte im weiteren Verlauf die Anzahl der Kommunikations- und Annäherungsversuche gegenüber der britischen Königsfamilie und Politikern sowie die Anzahl der Polizeieinsätze reduziert werden konnte. Beyli-Helmy et al. (2020) schlugen für die Evaluation eines erfolgreichen Bedrohungsmanagement vor, folgende Kriterien zu analysieren, die nicht nur die empirisch schwer zu erfassende Verhinderung von schweren Gewaltstraftaten abbilden:

- Der Informationsfluss ist gewährleistet (Bewegung ins Hellfeld).
- Kontakt mit den Betroffenen wurde hergestellt (z. B. Gefährder, Gefährdete, Behörden).
- Ein stringentes Fallverständnis wurde erarbeitet.
- Gefährder erscheinen zu Terminen und sprechen ihre Probleme an.
- Die besprochenen (Notfall‑)Strategien funktionieren (z. B. Situation verlassen; anrufen, bevor man in Versuchung kommt, beispielsweise ein Kontakt- und Rayonverbot zu missachten).
- Relevante Fachpersonen wurden eingebunden oder der Kontakt hergestellt (z. B. Fachstellen, Therapie).
- Betroffene Personen/Teams/Behörden werden entlastet (z. B. durch Verantwortungsteilung oder Akzeptanz der Unveränderbarkeit einer Situation).
- Die Vorfallsfrequenz nimmt qualitativ (Schwere) und quantitativ (Häufigkeit) ab.
- Grenzen des Machbaren werden akzeptiert.

Das Unterfangen, die Präventionsarbeit des KBM empirisch sichtbar zu machen, wird im Kanton Zürich weiter vorangetrieben. So hat eine extern in Auftrag gegebene Untersuchung ergeben, dass die Arbeitspartner im KBM Zürich mit den Strukturen und Prozessen sehr zufrieden sind. Verbesserungspotenzial wird z. B. an den Schnittstellen zum Gesundheitssystem verortet (Biberstein et al. 2020). Auch von den betroffenen Gefährdern werden die Tätigkeiten des Gewaltschutzes und der FFA angenommen: Die im Kontext des Gewaltschutzes kontaktierten Gefährder lassen sich in mehr als 80 % der Fälle auf ein oder mehrere Gespräche mit dem Gewaltschutz und der FFA ein (persönliche Rückmeldung der Gewaltschutzdienste, basierend auf interner Statistik 2020; Beyli-Helmy et al. 2020). Dieser persönliche Kontakt ist von hoher Bedeutung, um eine differenzierte Risikoeinschätzung zu erhalten und konkrete Unterstützungsmöglichkeiten mit den Gefährdern zu besprechen.

## Fazit

Das Zusammenspiel von polizeilichem und forensischem Fachwissen im frühen Fallstadium hat sich in der Praxis gerade auch im Hinblick auf die Entwicklung eines gemeinsamen Fallverständnisses und bei Gefährderansprachen bewährt. Die Beratung und der konkrete Einbezug von am Fall beteiligten Fachpersonen und Institutionen sind wichtig. Das Bedrohungsmanagement muss seine Arbeitsweise transparent darlegen und seine Vorgehensweise stets kritisch hinterfragen. Letztlich ist aber zu akzeptieren und aktiv zu kommunizieren, dass auch bestmögliche gewaltpräventive Bedingungen nicht alle Gewalttaten verhindern können. Durch ein professionelles Bedrohungsmanagement wird ein Beitrag geleistet, um menschliches Leid zu reduzieren. Dieser Beitrag kann u. a. darin bestehen, Menschen, die sich im Gefolge von Konflikten mit Behörden oder innerhalb von Beziehungen in eine destruktive Dynamik verstrickt haben, wieder zu konstruktiverem Verhalten zu ermutigen und sie auf diesem Weg auf eine niederschwellig-pragmatische Art zu unterstützen.

## Footnotes

1 Definition gemäß Auszug aus Dienstanweisung für Gefährderansprachen, Kantonspolizei Zürich, Präventionsabteilung (2020). Mit freundlicher Genehmigung durch R. Brunner.
